# The n–Si/p–CVD Diamond Heterojunction

**DOI:** 10.3390/ma13163530

**Published:** 2020-08-10

**Authors:** Szymon Łoś, Kazimierz Paprocki, Mirosław Szybowicz, Kazimierz Fabisiak

**Affiliations:** 1 Institute of Physics, Kazimierz Wielki University, Powstańców Wielkopolskich 2, 85090 Bydgoszcz, Poland; paprocki@ukw.edu.pl (K.P.); kfab@ukw.edu.pl (K.F.); 2 Faculty of Materials Engineering and Technical Physics, Poznań University of Technology, Piotrowo 3, 60965 Poznań, Poland; miroslaw.szybowicz@put.poznan.pl

**Keywords:** heterojunction, CVD diamond, defects dependent current, cathodoluminescence spectra, IV characteristic

## Abstract

Due to the possible applications, materials with a wide energy gap are becoming objects of interest for researchers and engineers. In this context, the polycrystalline diamond layers grown by CVD methods on silicon substrates seem to be a promising material for engineering sensing devices. The proper tuning of the deposition parameters allows us to develop the diamond layers with varying crystallinity and defect structure, as was shown by SEM and Raman spectroscopy investigations. The cathodoluminescence (CL) spectroscopy revealed defects located just in the middle of the energy gap of diamonds. The current–voltage–temperature, I−V−T characteristics performed in a broad temperature range of 77–500 K yielded useful information about the electrical conduction in this interesting material. The recorded I−V−T in the forward configuration of the n–Si/p–CVD diamond heterojunction indicated hopping trough defects as the primary mechanism limiting conduction properties. The Ohmic character of the carriers flux permitting throughout heterojunction is intensified by charges released from the depletion layer. The magnification amplitude depends on both the defect density and the probability that biasing voltage is higher than the potential barrier binding the charge. In the present work, a simple model is proposed that describes I−V−T characteristics in a wide range of voltage, even where the current saturation effect occurs.

## 1. Introduction

The field of CVD diamonds is rapidly maturing into one wherein new applications and products are being developed [1,2]. It is a currently emerging technology with immense promise for innovative, convenient, and high-performance electronics. It is related to the properties of this material, such as the wide bandgap, high breakdown voltage, high thermal conductivity, small dielectric constant, and excellent radiation hardness [3,4]. In connection with the above, diamond heterojunction structures can be recognized as a suitable material for the electronic devices which can operate at high temperatures and/or in chemically harsh environments. To better and more effectively use the potential of this material, many problems are yet to be solved. In particular, the electrical properties of diamonds, including those of Schottky contacts, are not well-known but are essential from potential future applications’ points of view. It is generally known that "as grown" diamond layers obtained by CVD methods show similar surface p-type conductivity regardless of whether they are doped or undoped [5,6]. It was also demonstrated that the high p-type conductivity of the diamond layer disappeared by oxidation but can be recovered by exposure to a hydrogen plasma or by cathodic pretreatment [7]. The diamond layers obtained by the HF CVD method are polycrystalline in nature and contain a high concentration of different defects, such as $sp^{2}$ phase admixtures, dislocations, vacancies, and dangling bonds. [8,9,10,11,12].For many semiconductors, it is known that the behavior of hydrogen is very complex, including the termination of dangling bonds, passivation of shallow and deep levels, and creation of extended defects [13,14]. In order to explain the high surface conductivity of diamonds, several models have been proposed. According to Landstrass et al. [15], the high conductivity layer is caused by passivation of deep levels by hydrogen incorporated in the subsurface region in the diamond layer. It was also suggested that the high-conductivity should be attributed to generation by included hydrogen; the shallow acceptor levels in the surface region [16] were also later confirmed by Hall measurements [17]. According to Kawarada et al. [18] hydrogen termination of the diamond’s surface makes it negatively charged, which induces a layer of hole accumulation near the surface, and it leads to band bending. Mechanisms of the hole generation are still not fully understood, and they remain controversial but detailed information about carrier transport and electronic states in the high-conductivity layers is necessary to be clarified.

In this paper, we present a new approach to the description of the mechanism of electrical conductivity in CVD undoped polycrystalline diamond layers. In the hopping mechanism, regarded as the primary one in the studied materials, the Schottky and Poole–Frenkel effects have to take place [19,20]. The electric charges freely diffusing from the silicon substrate through the interface to the carbon diamond structure are localized due to the rising potential of the depletion layer and the potential associated with the defects. These two phenomena influence the current permitted through the heterojunction differently. In a low voltage region, the current density is governed by the Schottky barrier; however, in higher voltage regions, the current can be magnified by charges released from defects sates due to Poole–Frenkel emission. In our approach, we propose the new model for description of the I–V’s characteristics, which allows us to calculate the characteristic parameters of heterojunction, such as the distribution of the potential barriers and the value of the total charge gathered on the interface responsible for magnifying the current.

## 2. Results and Discussion

### 2.1. The SEM Micrographs Morphologies

**Figure 1:**
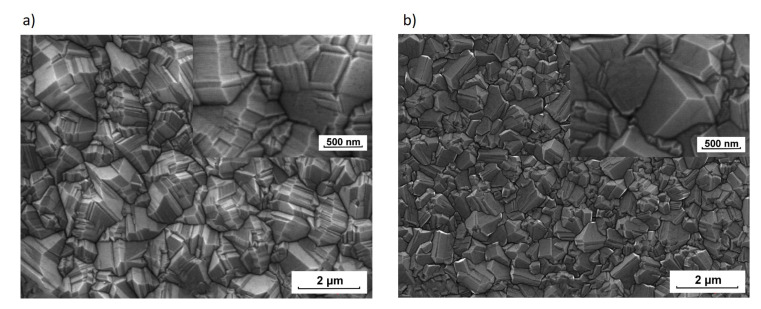
SEM micrographs of the diamond layers synthesized with different working gas compositions: (**a**) DFI CH_3_OH/H_2_ = 1.0 vol.%, (**b**) DFII CH_3_OH/H_2_ = 4.0 vol.%.

The morphologies of diamond samples were observed by scanning electron microscopy. The SEM micrographs, presented in the Figure 1, display surface morphologies of two diamond films DFI and DFII produced under different working gas compositions. Both images show an apparent feature of diamond crystallites. However, Figure 1a shows morphology indicating the (111) orientation of diamond microcrystals. The morphology seen in Figure 1b has mainly a mixed character. Such statements seem to be confirmed by a detailed analysis of the inserts in the upper right corners of the photos in Figure 1.

**Figure 2:**
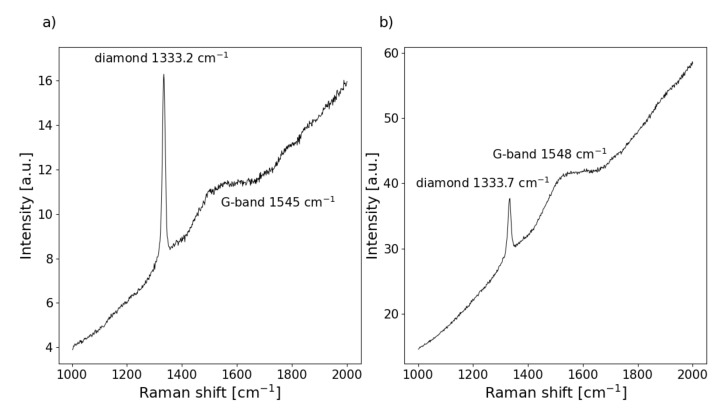
The Raman spectra of the studied films (**a**) DFI and (**b**) DFII.

The morphological and structural differences of diamond films are reflected in their Raman spectra. For both samples, spectra (Figure 2) show the relatively strong diamond Raman peaks at around 1333 cm${}^{-1}$ and brad G–band at 1545 cm${}^{-1}$. They were superimposed on a broad luminescence background. The full width at half maximum, FWHM, is one of the most important parameters for describing a diamond’s structural quality. This factor describes the phonon-free path or lifetime. At room temperature the main mechanism responsible for phonon scattering involves structural defects introducing anharmonic effects [21,22]. If anharmonic terms are taken into account in the lattice Hamiltonian, the phonons are no longer eigenstates of the system, and they become finite-lived quasiparticles described by a complex self-energy, which in turn determines the line shape of the associated Raman peak [23,24]. Based on the work of P.M. Fauchet et al. [25], it was shown that there is a simple relationship between the FWHM and phonon-free path length, L. The FWHM × L $\approx 90\;[{\mathrm{cm}}^{-1}\mathrm{nm}]$ [26]. The FWHM has to be expressed in cm${}^{-1}$ and L in nm. If we assume that the phonon-free path corresponds to the average distance between defects, their concentration N${}_{d}$ can be easily estimated by the following formula: N${}_{d}∼$ L${}^{-3}$. The FWHMs of the diamond Raman peaks presented in the Figure 2 are 11.3 cm${}^{-1}$ and 15.6 cm${}^{-1}$ respectively, which in turn correspond to defect concentrations of $2.1\times 10^{18}$ cm${}^{-3}$ and $5.3\times 10^{18}$ cm${}^{-3}$ respectively for sample DFI and DFII. The other important feature derived from the Raman spectrum is the level of hydrogen termination of the diamond surface [6]. This can be read from a luminescence background slope according to the empirically-derived equation [27]. Despite an apparent contrast between slopes, the calculated hydrogen concentration is equal for both samples. Its value is about 18 vol.%. This is due to variation of the D-band to G-band intensity ratio as one important factor in the formula.

### 2.2. The Cathodoluminescence Spectroscopy

**Figure 3:**
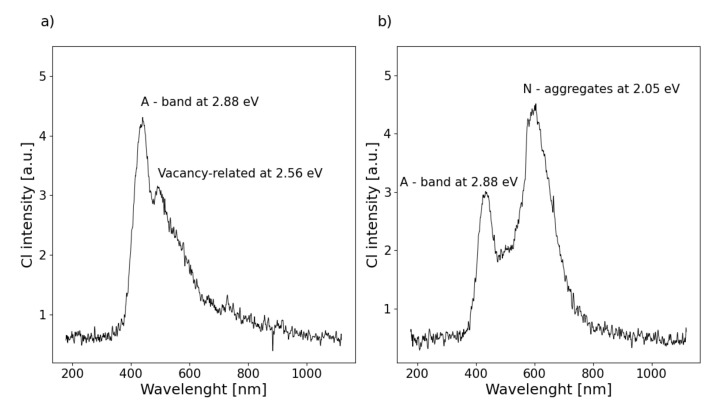
Cathodoluminescence spectra for morphologies of (**a**) DFI and (**b**) DFII diamond layers.

CL spectroscopy has been recognized as a very sensitive and non-destructive analysis method, which can provide valuable information about the nature of defects, Figure 3.

In general, typical CL spectra of CVD diamond films are dominated by a broadband in the blue spectral region (A-band), which is observed for almost all diamond types [28,29,30]. Some models of the origins of band-A emissions have been reported. The most widely used model is based on the donor–acceptor pair recombination mechanism [31]. The above model is based on randomly distributed boron as the acceptor. Nevertheless, in the case of undoped diamond films, this model can not be applied. According to M. Marinelli et al., the band-A emission from undoped CVD diamond layer can be attributed to lattice disorder such as dislocations [32]. Since the band-A emission behavior in homoepitaxial CVD diamond films was the same as that in polycrystalline films, this suggestion takes into account all the sp^2^ defects in a model of the origin of band-A emission [33]. Our earlier work showed that the CL spectra of the CVD diamond layers strongly depend on the morphology and the preferred orientation of diamond microcrystallites [12]. Among the materials synthesized under varying conditions, there can be distinguished two types of spectra. They are presented in Figure 3. It should be noted that both synthesized layers types the DFI and the DFII showed similar radiation recombination centers in positions 2.88 and 2.56 eV, but in the second sample, an additional recombination center appeared at 2055 eV [34,35]. These defects were due to nitrogen incorporated into synthesized material from residual gas in the reaction chamber.

### 2.3. The Electrical Properties

**Figure 4:**
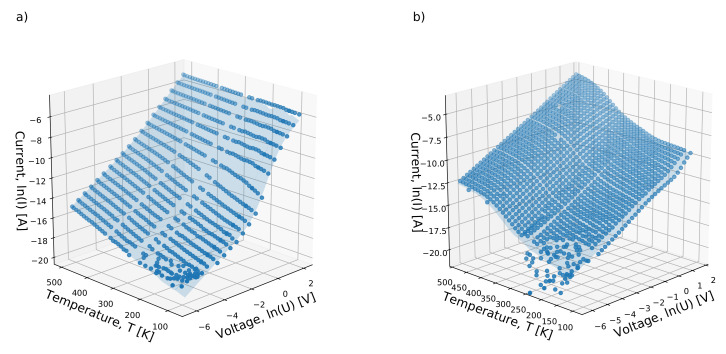
The gathered I−V−T characteristics in the forward configuration plot versus the temperature and the voltage for both developed heterojunctions (**a**) DFI and (**b**) DFII with a calculated hypothetical conducting surface.

The whole set of gathered data in the forward direction for developed heterojunctions with different morphologies of diamond layer has been presented in Figure 4.

**Figure 5:**
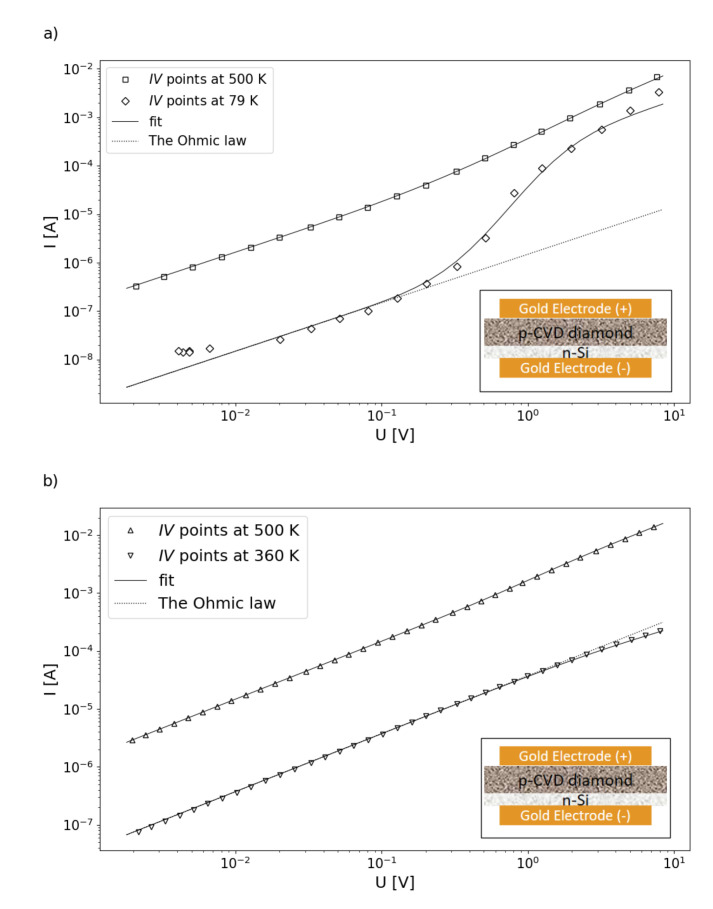
Details of I−V−T characteristics for selected temperatures of (**a**) DFI and (**b**) DFII developed heterojunctions respectively. Insets: the scheme of the heterojunction structure.

**Figure 6:**
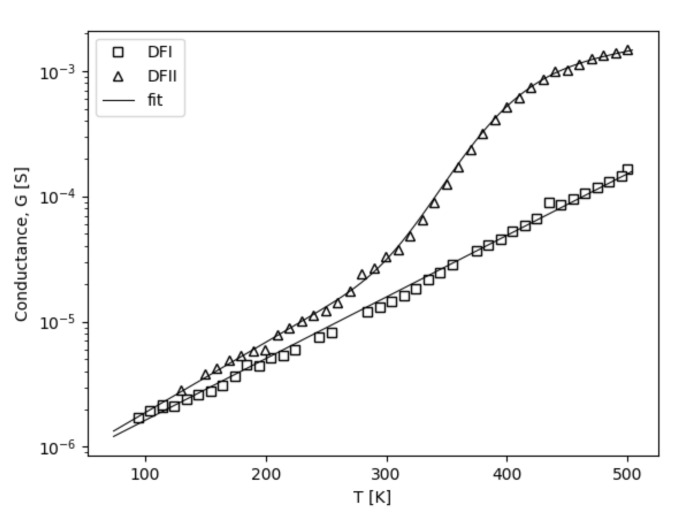
The temperature dependencies of the conductance *G*.

There are easily-seen distinctions between the I−V−T characteristics, despite both experiments having recorded a similar number of points. The second heterojunction based on the DFII diamond layer better conducts the current, especially in a higher temperature region, as the measured current has higher values. On the opposite side of the temperature and the low biasing voltage range, both heterojunctions permit only a leakage current, as they are blocked by the built-in potential. The differences observed in the CL spectra are reflected in the electrical properties. Through the DFI heterojunction, characterized by the strong A-band at 2.88 eV and the valency-related band at 2.56 eV in the spectra, Figure 3a, the whole temperature range flows with exponentially increasing current. For distinctness, in Figure 5a are shown I−V−T characteristics with a different magnification amplitudes factor for selected temperatures. In the case of the DFII layer, the conversion of the magnification factor was observed, from a positive one at 500 K to negative at 360 K, Figure 5b. Finally, at a low-temperature range, the magnification effect takes place again. To describe changes in the I−V−T characteristic in the forward configuration, we propose a new approach that differs from the one commonly used in the literature [36,37]. In the whole voltage range, the I−V−T can be designated by the following formula:

(1) $$I=GU\exp\left(A\int_{0}^{U}f\left(U_{0}\right)dU\right),$$

where *G*—conductance, *U*—biasing voltage; *A*—magnification factor; $P\left(U\geq U_{0}\right)=\int_{0}^{U}f\left(U_{0}\right)dU$ represents probability, with: $f(U_{0})$—distribution function and $U_{0}$—a point at which cumulative distribution function takes value of 0.5 and biasing voltage *U* is higher or equal to potential localizing charges in heterojunction area. This simple model can be applied to entire voltage range. For several milivolts, where the probability $P(U\geq U_{0})\approx 0$, the Ohmic behavior is observed. Additionally, the current fluxes in both directions are of the same order. In a higher voltage regime though, where the $P(U\geq U_{0})\approx 1$, the saturation effect takes place, possibly caused by Poole–Frenkel emission. The *A* parameter together with the $P(U\geq U_{0})=1$ gives a total accessible number of states at any temperature condition at the heterojunction. Taking into account the accuracy of calculations, it was determined that the $f(U_{0})$ has the shape of a log-normal distribution function with a width of δ—parameter. Results of the calculation procedure are visualized in the Figure 4 by the hypothetical surface of conduction plot and in the Figure 5 as a solid line for chosen temperatures as well. The Equation (1) fits well with experimental points as a correlation coefficient; *r* is close to 0.9998. A negative value of the *A* parameter introduced in Equation (1) describes the odd current decrease at higher voltage observed for the DFII sample at the temperature of 360 K, Figure 5b. It should be noted that parameters *G*; *A*, an average value of localizing potential height; ${\bar{U}}_{a}$; and δ in Equation (1), are temperature dependent. CL spectra reveal the presence of a defect band located in the middle of the diamond gap. The *A* parameter, together with the $f(U_{0})$ function, allowed us to investigate the localization strengths of charges. The nature of the created defect band revealed the CL spectra. They were dislocations forming A-band at 2.88 eV and vacancy-related states at 2.56 eV both created during the synthesis procedure, Figure 3a. Additionally, Raman spectra analysis has been allowed to estimate their average density. A lowering of the *A* value with temperature increase led to a decrease of defect number, which strongly localized electrons, since at the same time, the average value of ${\bar{U}}_{a}$ was increasing. For lower temperature in the vicinity of 78 K the *A* took the value of 5, and the mean value of potential wall height ${\bar{U}}_{a}$ was 0.968 V with the δ = 0.727 distribution width. However, in the higher temperature range, i.e., 500 K, the above parameters took values respectively of 1.92, 5.69 V, and 1.65. Due to carrier’s higher thermal energy, the magnification effect had a smaller amplitude as an activation process approached the "band top" created by defects, where there werea few states with high values of localizing potential. Out results clearly show the current magnification permeating through the heterojunction. Charges are gradually released from localized states associated with structural defects of the diamond lattice by the high enough biasing. A proper forward voltage polarization of the heterojunction indicates lower potential value on the diamond site [38]. This results in free electrons’ diffusion from Si to CVD diamond and forming of the depletion layer, that being the basic charge repository responsible for current reinforcement. However, the biasing potential increased releases charges from deeper states and ultimately saturated the current due to the Poole–Frenkel emission effect. The most important parameter derived from the Equation (1) is the thermal dependency of the conductance, *G* presented in Figure 6.

Similarly, like for iron perovskites crystal [39] the conductance rise with temperature increases, probably, due to a lack of current carries interactions with phonons. In the case of the DFI sample the conductance increased purely exponentially. It was caused by the increase of carrier concentration with temperature. However, for the DFII, a little above-room temperature extra activation process is perceived. As before, that leads to the saturation effect at high temperatures. If the temperature scale can be considered as the energy scale, we propose to describe the temperature dependence of the conductance by the following equation:

(2) $$G=G_{0}\exp\left(\frac{T}{T_{0}}+A^{{}^{\prime }}\int_{0}^{T}f\left(T_{0}^{{}^{\prime }}\right)dT\right).$$

Due to the fact that graphs of the *G* versus temperature are convergent for both diamond samples, the $G_{0}$ and $T_{0}$ parameters can be interpreted as heterojunctions of the materials’ constants. The $G_{0}$ parameter is given by formula:

(3) $$G_{0}=n\mu \frac{S}{l}$$

**Figure 7:**
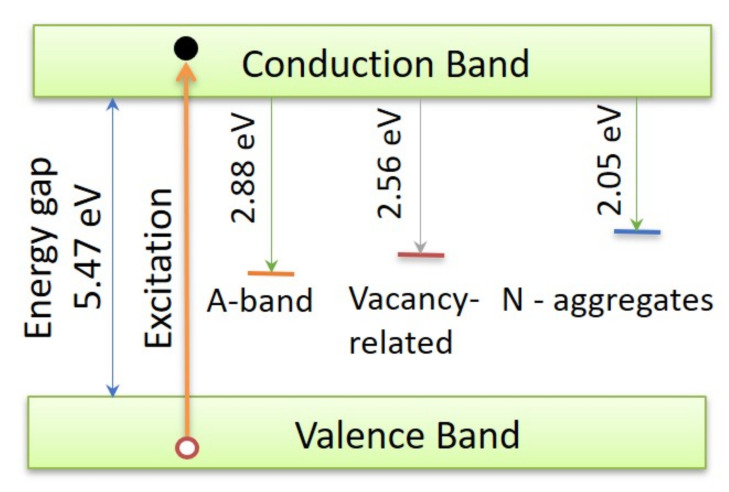
The scheme of radiative recombination centers in the band gap of CVD diamonds.

It is a product of: *n*—carrier concentration, μ—carrier mobility, *S*—the area of the heterojunction, and *l*—diamond layer thickness, 3–4 μm. It was found that $n\mu =46.24\times 10^{-9}\frac{S}{m}$. Taking into account a high value of electron mobility $\mu =0.21\frac{m^{2}}{Vs}$[38] gives the *n* parameter equal to $1.38\times 10^{15}\frac{electrons}{m^{3}}$, whereas the $T_{0}$ determines the slope of the G versus temperature plot. Usually, this parameter is interpreted as the activation energy of the conduction mechanism. It describes the energy gap or just a band bottom created by defects. For synthesized materials, it takes the value of only 7.5 meV. Therefore, to have the conductivity effect, it is necessary to fulfill at least one of two conditions: the heterojunction has to be at a higher temperature than $T_{0}$ = 82.78 K or the biasing voltage has to be higher than 7.5 mV. Otherwise, almost constant current controlled by built-in potential permeates the heterojunction. This behavior is demonstrated by odd points beyond the fitting line of I−V−T curves in Figure 5a gathered at 79 K in low voltage range or beyond the surface in Figure 4. This specified conduction mechanism is called as a trap filling regime [40]. It vanishes with the increase of the biasing voltage or the temperature. Such a low value of the activation energy was attributed to the effect of space charge accumulation in the depletion layer [6]. These studies showed that electrons diffuse through the heterojunction from n-type Si to the p-type CVD diamond, and defect states trap them. The scheme of defects is shown in Figure 7.

The heterojunction biasing at temperatures higher than 82.78 K cause a hopping mechanism to realize carrier flux. It is strengthened by electrons released from traps. With the temperature rise, the magnification effect is smaller as the number of accessible states decreases with the energy increase. An almost straight line is observed at the log scale of the I−V−T characteristic, Figure 4. For the DFII heterojunction, another density of states with higher energy appeared; see Figure 6. It is the layer in which the N-aggregates peak at 2.05 eV [34,35], which is shown on the Cl spectrum. In this case, the conductance increase can be described by a second term of the exponential function in Equation (2). As before, it represents the probability—with a distribution function, $f(T_{0}^{{}^{\prime }})$ taking the shape of the log-normal form as well, while the ${\bar{T}}_{a}$ is an average value equal to 429.92 K (37 meV) and the magnification factor is $A^{{}^{\prime }}$ equal to 1.86—that the thermal energy is higher or equal to the height of potential responsible for the charge localization. The presence of these states results in the material’s conductance increasing. That was confirmed by the higher values of average defect concentration derived form Raman spectroscopy. The activation process starts at room temperature; thermal energy causes charge to be released from nitrogen traps. [41]. However, the same current limitation effect takes place, as is visible on the I−V−T curve registered at 360 K; see Figure 5b. The negative value of the *A* coefficient in Equation (1) equal to −0.878 results from internal repulsion interaction between charges in A-band and N-aggregates. Higher thermal energy overcomes this interaction, and the Ohmic character was observed over 500 K.

## 3. Materials and Methods

The polycrystalline, undoped diamond samples were synthesized in the form of thin (4–5 μm) layers deposited on n-type (100) oriented silicon wafer substrates. The electrical conductivity of substrates was tested by the point probe FPP5000 Veeco Intrument inc. The synthesis was performed in a hot filament CVD reactor (HF CVD), using methanol vapor diluted in hydrogen (CH_3_OH/H_2_ = 1.0–4.0 vol.%). The other technological parameters were as follows: total pressure in the reaction chamber—60 mbar, substrate temperature—1000 K, and gas flow rate—100 sccm (standard cubic centimeter per minute). Before diamond synthesis, the substrate was subjected to the standard procedure necessary for enhanced diamond nucleation density, i.e., mechanically polished with diamond paste and then washed in the ultrasonic bath in methanol and acetone.

The Raman spectroscopy was used for structural characterization of the diamond samples. The spectra were recorded at room temperature in back scattering geometry using Renishaw in Via Raman spectrometer. A 488 nm line from a tunable Ar ion laser was used as an excitation source. The Raman scattering spectra were recorded in the range of 1000–2000 cm${}^{-1}$. All data collection were analyzed using Renishaw WiRE 3.1 software using curve fitting method.

For diamond layer morphology the scanning electron microscope Jeol type JSM–820 operating at 25 kV was used. The cathodoluminescence, CL analysis was performed using FE-SEM apparatus modified in order to take CL spectra. The CL emission spectra were registered at room temperature using a grating spectrometer equipped with a charge-coupled device camera. The accelerating voltage of the electron beam was 30 kV. Two gratings were used, giving a spectral resolution of 0.5 nm or 0.2 nm in the case of high resolution spectra. The magnification was varied between 10 and 40 k and the acquisition time was between 30 and 60 s.

The I−V−T curve characteristics were recorded using following instruments: Rigol DG1022A as the power supply generating a rectangular voltage wave with peak to peak amplitude in the range of 4–20 V. It was working with frequency of 0.1 Hz, which is proper value for gathering measurements of: a current flowing with a Keithley instrument of a series 6485 picoammeter and a potential drop in the sample with Fluke 8505A Digital Multimeter device. The temperature was stabilized by Mercury controller as the measurements were carried out in temperatures from 77.4 to 500 K in Optistat cryostat. Both instruments were produced by the Oxford Instruments company. In order to measure the I−V−T characteristic, the samples’ surfaces were metalized with a gold electrode to ensure a proper electrical contact to a material on the substrate side and on diamond layer as well. Before starting the I−V−T measurements the cleaning procedure consisted of heating the sample to 500 K in low pressure inside of the cryostat. Above procedure is necessary because of the diamond surface sensitivity to adsorbed molecules.

The calculation procedure was carried out using Python language with the visualization Matplotlib package [42].

## 4. Conclusions

The structural studies using SEM imaging together with Raman spectroscopy indicate that defects and the hopping mechanism are responsible for the conducting properties on developed heterojunctions. The analysis of the CL spectra showed the presence of the defect band inside of the diamond gap. They were mainly due to dislocations, were vacancy-related, or were N-aggregates. It was shown that changes within the energy band gap have an essential influence on the I−V−T characteristic. The proposed new approach to the I−V−T description allows us to determine the localization strength of charges in the depletion layer, and we found some important material heterojunction constants, such as charge concentration and energy activation of the conduction mechanism.

## Abbreviations

The following abbreviations are used in this manuscript:

I−V−T	the current-voltage-temperature charcteristic
I−V	the current-voltage characterstic
HF CVD	Hot Filament Chemicla Vapor Deposition
SEM	Scanig Electrton Microspoy
CL	the cathodoluminescence
FWHM	the full width at half maximum
